# First experimental demonstration of magnetic resonance-guided multileaf collimator tracking for (ultra-)hypofractionated prostate radiotherapy^☆^

**DOI:** 10.1016/j.phro.2025.100828

**Published:** 2025-09-13

**Authors:** Prescilla Uijtewaal, Pim T.S. Borman, Peter L. Woodhead, Hans C.J. de Boer, Bas W. Raaymakers, Martin F. Fast

**Affiliations:** a Department of Radiotherapy, University Medical Center Utrecht, The Netherlands; b Elekta AB, Stockholm, Sweden

**Keywords:** Prostate cancer, MLC-tracking, Plastic scintillation dosimetry, MR-linac

## Abstract

**Background and purpose:**

(Ultra-)hypofractionated radiotherapy is an effective treatment for localized prostate cancer, but intrafraction motion can increase toxicity and/or reduce treatment efficacy. Therefore, motion management is essential. This study explores magnetic resonance imaging (MRI)-guided multileaf collimator (MLC) tracking for 2-fraction prostate radiotherapy on an MR-linac.

**Materials and methods:**

We compared two MRI-guided MLC centroid tracking workflows, each using a different motion manager to derive and stream target positions to our in-house MLC tracking software. The first workflow relies on interleaved 2D (2.5D) cine-MRI, introducing minimal latency. In contrast, the second workflow utilized 3D cine-MRI, which operates at a relatively lower imaging frequency that introduces more latency.

For experimental validation, we used a motion phantom equipped with an integrated insert that combines film with plastic scintillation dosimetry. A 2x12 Gy 11-beam prostate intensity modulated radiotherapy plan was created for tracking deliveries.

**Results:**

The signal latency introduced by the motion managers was 0.6 s for 2.5D cine-MRI and 6.3 s for 3D cine-MRI. Despite this latency, MLC tracking effectively restored the planned dose, improving the 2%/2mm local gamma pass-rates from 21% (due to linear drift) to 89% (2.5D) and 91% (3D). Plastic scintillator measurements showed reduced dose deviations at the periphery of the clinical target volume from 13–64% (no tracking) to 0–11% (2.5D) and 2–26% (3D).

**Conclusion:**

Our experiments demonstrated the technical feasibility of 2.5D and 3D cine-MRI-based MLC tracking on an MR-linac for 2-fraction prostate radiotherapy, with both motion management strategies achieving comparable dosimetric improvements despite the difference in latency.

## Highlights

• 3D multileaf collimator tracking for magnetic resonance guided prostate radiotherapy.
• Tracking has clear dosimetric advantages for prostate stereotactic body radiotherapy.
• A novel, time-resolved, motion-included, and magnetic resonance compatible dosimeter.

## Graphical abstract

**Figure:**
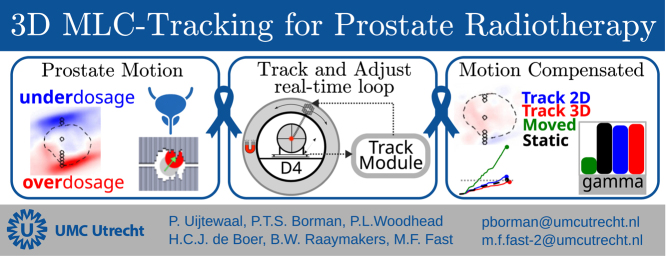

## 1 Introduction

Stereotactic body radiotherapy (SBRT) is an effective and safe treatment for low- and intermediate-risk prostate cancer [1]. Prostate cancer has a high intrinsic sensitivity to fractionation (low α/β ratio) [2], [3], increasing the desire for (ultra-)hypofractionation, which typically means treating in 2–5 fractions. However, implementing ultra-hypofractionated prostate SBRT poses significant challenges, particularly due to intrafraction prostate motion. This motion is mainly caused by bladder [4] and rectal filling [5], muscle tension [6], and posture changes [7]. The likelihood and magnitude of motion increases during ultra-hypofractionated treatments, as longer beam-on times allow for greater motion and deformation [8], [9], [10]. Although the impact of intrafractional motion on toxicity and local control in hypofractioned prostate SBRT and therefore its clinical significance is not yet well established, any such motion is likely to complicate precise prostate targeting. This increases the risk of radiation exposure to surrounding organs at risk (OARs), which potentially leads to higher toxicity and/or reduced treatment efficacy.

To safely implement ultra-hypofractionated prostate SBRT, active motion mitigation strategies are essential [8]. The introduction of a linear accelerator integrated with a magnetic resonance imaging (MRI) scanner (MR-linac) offers new opportunities to reduce the dosimetric effects of intrafraction motion [11], [12]. One such approach is sub-fractionated treatment workflows, which have been introduced for prostate radiotherapy [13], [14] and are currently being evaluated for 2-fraction SBRT in the HERMES clinical trial [14]. Additionally, automatic beam gating and baseline shift corrections are clinically available on MR-linacs [15], [16], [17]. While these techniques effectively mitigate prostate motion, they might do so at the expense of treatment delivery efficiency [18]. A more efficient alternative may be multileaf collimator (MLC) tracking.

The benefits and feasibility of MLC centroid tracking for prostate SBRT on C-arm linacs have been well established in literature [19], [20], [21], and have even been demonstrated in a clinical trial [22]. The technical feasibility of MRI-guided MLC centroid tracking has already been demonstrated on an MR-linac [23], [24], where tumor translations were derived from a research-based 2D cine-MRI sequence. However, this sequence lacks robustness against out-of-plane motion, which could result in anatomical structures to intermittently enter or exit the field of view. As a solution, the clinical MR-linac systems are now equipped with a vendor-provided motion manager that continuously monitors translational 3D tumor motion using orthogonal interleaved 2D (2.5D) cine-MRI [15], [16]. While originally designed to support automatic beam gating, the generated 3D motion vector might also be used for MLC tracking.

Another approach to avoid out-of-plane motion is the use of 3D cine-MRI. Although 3D acquisition times are generally considered too slow for real-time applications due to concerns about latency, leading most to prefer 2.5D cine-MRI for motion tracking, this limitation primarily impacts the tracking of rapid, respiration-induced motion [25]. However, prostate motion is minimally affected by respiration [26] and is instead dominated by relatively slow drift motion [5], [8], which can be effectively tracked with 3D cine-MRI [8]. Additionally, 3D imaging provides motion data for the entire anatomy, enabling precise (retrospective) dose accumulation [27], [28]. Despite these advantages, 3D cine-MRI has yet to be integrated into an online workflow for active motion mitigation.

This study introduces two MRI-guided MLC tracking workflows on an MR-linac, utilizing either 2.5D cine-MRI guidance or 3D cine-MRI guidance to mitigate intrafractional anterior-posterior (AP) and cranial-caudal (CC) prostate translations. Accumulated dose distributions and time-resolved point dose measurements of both approaches will be evaluated and compared to assess their effectiveness in motion mitigation.

## 2 Materials and methods

### 2.1 MLC tracking implementation and workflows

**Fig. 1:**
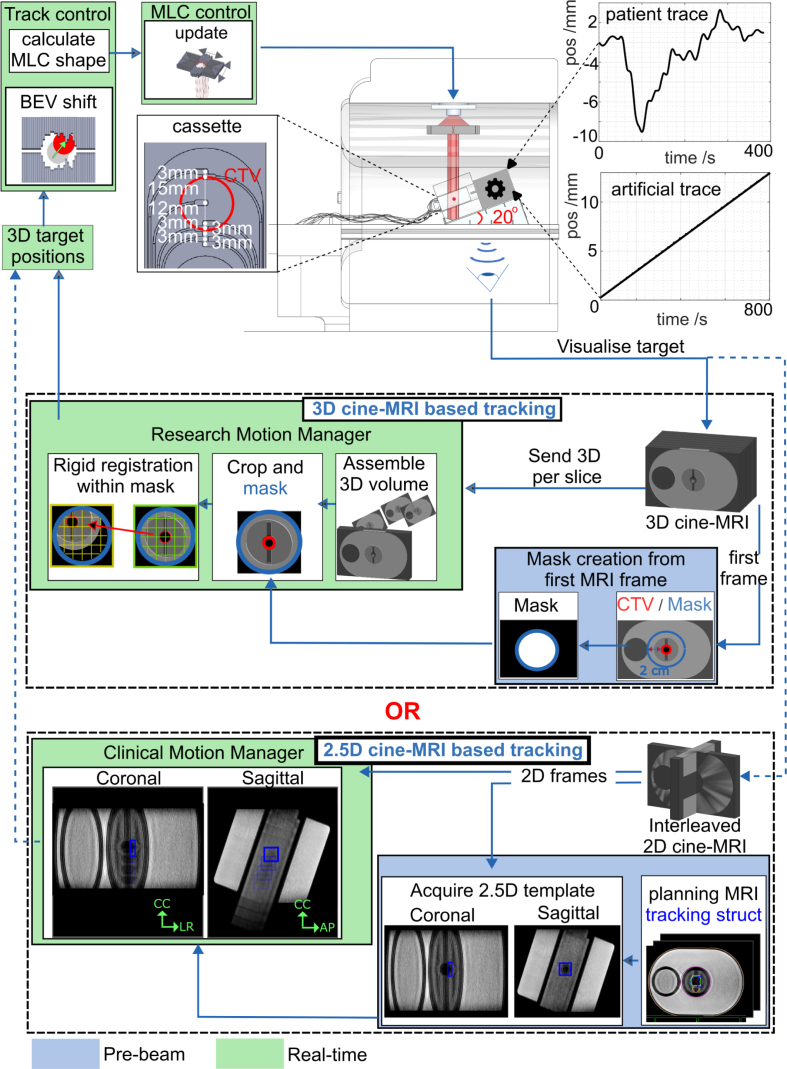
Experimental setup for a 3D and 2.5D MRI-cine based tracking workflow. Each workflow used either the 3D cine-MRI-based motion manager or the 2.5D cine-MRI-based motion manager. The prototype hybrid dosimetry cassette was used for dosimetric evaluation.

All experiments were performed on a 1.5 T Unity MR-linac (Elekta AB, Stockholm, Sweden) in research mode, featuring an MLC with 160 leaves moving in CC-direction and two diaphrams moving perpendicular to them. To correct translational target motion, we developed and compared two MR-guided MLC tracking workflows (Fig. 1). Each workflow contained either a 2.5D or 3D cine-MRI-based motion manager, which continuously extracted the target position from real-time cine-MRI, as described below. In both workflows, a tracking controller first calculated the target shift in the beam’s-eye-view, then adapted the planned MLC aperture, and lastly, instructed the MLC to assume its new position.

### 2.2 Motion managers

We compared MLC tracking performance using workflows with either a clinically available 2.5D cine-MRI motion manager or an in-house-developed 3D cine-MRI motion manager.

#### 2.5D cine-MRI-based motion manager.

For the 2.5D cine-MRI-based workflow (Fig. 1), we used the Unity MR-linac’s comprehensive motion manager (CMM) – clinical software that tracks 3D target motion using interleaved 2D coronal and sagittal cine-MRI images to support automatic gating and baseline shift plan adaptations [15], [16]. Here we used a T2-weighted turbo spin echo cine-MRI sequence tailored for improved prostate visibility, that was recently clinically released into CMM [29]. The 2.5D cine-MRIs were acquired at 0.5 Hz per plane with a field-of-view (FOV) of 436 × 436 mm^2^, a voxel size of 2 × 2 × 5 mm^3^, a repetition time (TR) of 2.0 s, an echo time (TE) of 0.15 s, an acquisition shot-length of 0.30 s, and a flip angle of 90°. A tracking structure was defined in the 3D planning MRI, and the orthogonal cine-MRI acquisition was automatically centered on this structure. A motion vector was derived by CMM using a template-based registration algorithm [30], using a pre-beam acquired template, and served as input for MLC tracking.

#### 3D cine-MRI-based motion manager.

The 3D cine-MRI-based workflow (Fig. 1) was adapted from a previous soft-tissue prostate tracking study [31]. Within this workflow, we used a 3D balanced gradient-echo cine-MRI sequence with an acquisition time per frame of 9.36 s (FOV: 452 × 452 × 99 mm^3^, acquisition voxel size: 2 × 2 × 2.2 mm^3^, TR/TE: 4.7/2.3 ms, flip angle: 50°, parallel imaging factor: 3.6 × 1.2, partial fourier factor: 0.8 × 0.7). Immediately after acquiring each 3D frame, it was reconstructed and automatically streamed to our motion manager using ReconSocket [32].

Image processing was automatically initiated as soon as the motion manager received data. A target delineation transferred from the planning MRI to the first cine-MRI dynamic was used as tracking structure. To reduce processing times, subsequent 3D frames were cropped around the mask region. The motion manager then determined the target’s translation by performing real-time rigid registration of the current cine-MRI dynamic to the first dynamic using the SimpleElastix image registration toolbox [33]. The resulting translation vector was subsequently used for MLC tracking.

### 2.3 Experimental setup

To validate the MLC tracking performance of both workflows, the QUASAR MRI^4D^ motion phantom (IBA QUASAR, London, ON, Canada) was used. The phantom consists of an oval-shaped body containing a cylindrical insert that can be translated along a 1D motion axis. The insert contains a ∅3 cm spherical clinical-target-volume (CTV), which was intersected sagittally by a prototype dosimetry cassette. To increase the CTV’s motion complexity, the phantom was positioned on a 20° ramp (Fig. 1b), tilting the phantom’s motion axis and thereby effectively decomposing the target’s movement into both CC and AP directions, which are linearly correlated. The phantom was used statically as reference, was programmed with linear drift motion (CC_motion-range_ = −12–0 mm, CC_speed_ = −0.9 mm/min, AP_motion-range_ = −4–0 mm, AP_speed_ = −0.3 mm/min), or patient-derived prostate motion (CC_motion-range_ = −3.1–8.5 mm, CC_average_ = 1.1 mm; AP_motion-range_ = −1.1–3.1 mm, AP_average_ = 0.4 mm).

### 2.4 Treatment planning

A planning CT (voxel size = 1 × 1 × 1 mm^3^) was acquired on the Brilliance Big Bore CT scanner (Philips Medical Systems, Best, The Netherlands). The target was delineated as CTV, and isotropic 2 mm CTV-to-PTV margins were used. A 2 × 12 Gy 11-beam IMRT prostate SBRT plan was created in the treatment planning system (TPS) Monaco 6.2 (Elekta AB, Stockholm, Sweden). Following our clinical practice, a 3D planning MRI (T2-weighted, voxel size= 1.3×1.3×2.0 mm^3^) was acquired and an adapt-to-shape plan adaptation was applied [34]. Following our clinical guidelines, the plan was calculated with a 3 mm grid size and 3% statistical uncertainty per control point.

### 2.5 Dosimetric evaluation

To dosimetrically validate the MLC tracking performance, a prototype hybrid dosimetry cassette was used, which combines eight HYPERSCINT plastic scintillation dosimeters (PSDs) (Medscint, Quebec, Canada) with gafchromic dosimetry EBTXD film (Ashland Inc., Bridgewater, NJ). This dosimetry insert is an extension of the commercial version with four PSDs [35]. The cassette consists of two rectangular panels with a total thickness of 6 mm. The thicker 4 mm panel contains eight embedded time-resolved PSDs (size=0.5×0.5×0.5 mm^3^), arranged in a straight line along the phantom’s translational axis. One PSD is fixated at the center of the CTV, while the remaining PSDs are fixated around its periphery. Each PSD was connected by a 20 m long optical fiber that connects the PSD to the HYPERSCINT RP200 readout platform. The time-resolved PSD dose was measured with an integration time of 0.2 s An EBTXD film was positioned between the two panels, allowing for simultaneous measurement of both the film and PSD dose. Small pins are integrated into three of the cassette’s corners to simplify co-registration of film and PSD doses.

The films were digitalized using an Epson Expression 1000XL flatbed scanner (Seiko Epson Corp, Nagano, Japan), and analyzed using in-house-developed software [24]. A 2%/2 mm and 3%/3 mm local gamma-analysis evaluated the correspondence between the TPS dose and the MLC tracking deliveries. Only pixels >10% of the maximum dose were included, following the AAPM TG-218 recommendations [36].

The PSD dose measurements were also compared to the TPS dose to evaluate the MLC tracking performance. To minimize discretization artifacts, the dose was re-calculated with the minimum grid spacing of 1 mm and 1% statistical uncertainty per control point. In addition, the TPS dose grid was interpolated using 3D cubic splines to match the 0.5×0.5×0.5 mm^3^ PSD size. To minimize the influence of setup uncertainties, the film dose was registered to the TPS dose using intensity-based registration. The PSD locations were transformed accordingly.

### 2.6 Latency handling

The minimal system latency depends on the signal latency (T_signal_) – defined as the time elapsed from acquiring the center of k-space to extracting the target position from the image – and the MLC adjustment time (T_MLC_) [37].

To measure T_signal_ in both MLC tracking workflows, the phantom was positioned directly on the treatment couch and programmed with sinusoidal CC motion (A_peak-to-peak_ = 20 mm,T=60 s). The phantom constantly reports the target’s actual position with negligible latency (<1 ms). A sine wave was fitted to the phantom-reported positions and the image-derived target positions; the phase shift between them yields (T_signal_) [38].

T_MLC_ was estimated by using the MLC to directly track the phantom reported positions (sin, A_peak-to-peak_ = 20 mm,T=4 s). The electronic portal imaging device captured the MLC aperture and the phantom’s target at a frequency of 30 Hz; and the phase difference between both entities yields T_MLC_ [23], [38].

## 3 Results

In the 3D cine-MRI workflow, T_signal_ was 6300 ms, whereas it was only 600 ms in the 2.5D-based workflow. T_MLC_ yielded a relatively smaller latency of 98 ± 1 ms.

**Fig. 2:**
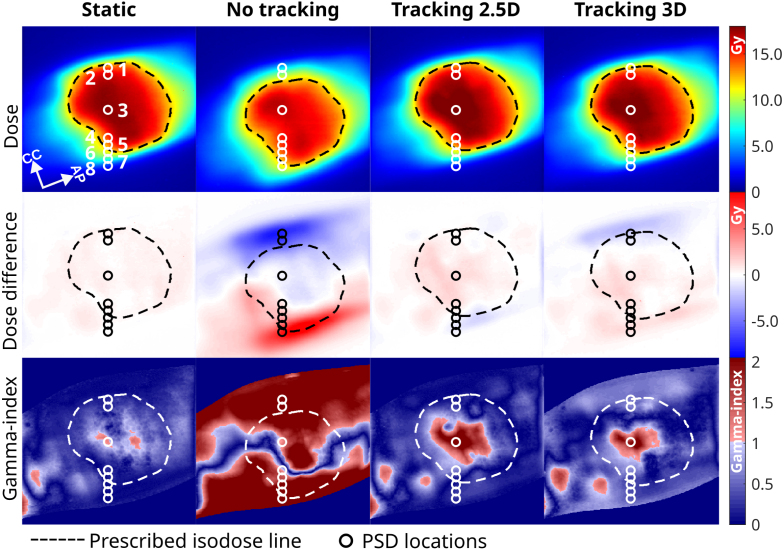
Dosimetric film results showing the absolute dose (1st row), dose difference with respect to the planned dose (2nd row), and local 2%/2 mm gamma maps (3rd row) for the varying motion compensation scenarios: no-motion reference (1st column), linear motion without MLC-tracking (2nd column), linear motion with 2.5D-based MLC-Tracking (3rd column), and linear motion with 3D-based MLC-Tracking (4th column). Note that in the 2nd column, the dose is shifted due to the absence of motion compensation.

Fig. 2 shows an overview of the dosimetric film results relative to the TPS dose for the case with linear drift motion. The treatment duration was approximately 13 min, regardless of MLC tracking mode. The dose difference maps highlight that, without motion compensation, maximum differences of 85% relative to the prescribed dose were observed. Applying 2.5D or 3D-based MLC tracking reduces these to 16% and 21%. This is supported by the local 2%/2 mm gamma pass-rates (Table 1), whereby both 2.5D and 3D-based MLC tracking improve the pass-rates from 21% without MLC tracking to 89% and 91%, respectively. Although patient-derived motion exhibited a relatively subtle effect – largely due to up-and-down drift fluctuations resulting in an 89% pass-rate without MLC tracking – the incorporation of 2.5D or 3D-based MLC tracking increased the pass rates to 99% and 96%, respectively. MLC tracking yielded pass-rates for both motion traces that agreed perfectly with the static reference’s pass-rate (98%). Importantly, both tracking workflows resulted in very similar pass-rates.

**Table 1:** The local gamma pass-rates for the different motion scenarios relative to the treatment planning system.

	Static		Linear drift motion						Patient-derived motion				
			No tracking		Tracking				No tracking		Tracking		
					2.5D cine		3D cine				2.5D cine		3D cine
Gamma pass-rates													
2%/2 mm (%)	98		21		89		91		89		99		96
3%/3 mm (%)	100		33		95		98		97		100		100

**Fig. 3:**
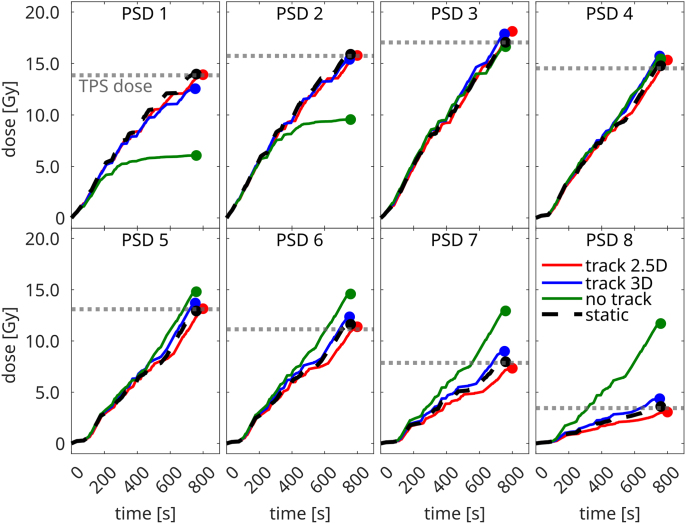
Time-resolved PSD results comparing the different MLC tracking workflows for linear drift motion.

Fig. 3 shows the time-resolved PSD measurements for linear drift motion. Most motion-induced dose differences were observed at the CTV’s periphery (PSD 1–2 & 5–7), where absolute differences relative to the TPS ranged from 13% to 64% (Table 2). In contrast, only modest differences (2%–6%) were observed within the CTV (PSD 3–4). Notably, PSD 8 – originally located in a low-dose region (Fig. 2) – experienced an even larger deviation when the drift moved it into a high-dose area, resulting in a delivered dose almost 2.5 times higher than intended. Both 2.5D and 3D-based MLC tracking effectively restored the TPS dose and reduced the absolute dose deviations to 0%–11% (Table 2) and 2%–26%, respectively. When tracking was applied the delivered dose was also very similar to the static case, with absolute differences ranging between 0%–15% (2.5D) and 3%–21% (3D). For all scenarios, the largest differences compared to the TPS were found in PSD 1 and PSD 7–8, where steep dose gradients (7-41%/mm), increased the uncertainty of the extracted TPS dose. Similar results were obtained for the patient-derived motion; however, as seen in the film measurements, the motion-induced dose differences were considerably more subtle. Note that the film and PSD dose agreed well with an absolute average difference of 1 ± 3%.

**Table 2:** Plastic Scintillation Dosimeter (PSD) dose as percentage difference relative to the TPS dose. Dose gradients [%/mm] indicate the dosimetric uncertainty for the extracted TPS dose.

	TPS			Static		Linear drift motion						Patient-derived motion				
						No tracking		Tracking				No tracking		Tracking		
								2.5D cine		3D cine				2.5D cine		3D cine
	(Gy)	(%/mm)		(Δ%)		(Δ%)		(Δ%)		(Δ%)		(Δ%)		(Δ%)		(Δ%)
PSD 1	13.85	7		1		−56		0		−9		−15		2		−2
PSD 2	15.74	2		1		−39		0		−2		−9		2		1
PSD 3	17.05	1		0		−2		6		5		−1		4		5
PSD 4	14.53	4		2		6		6		8		10		3		1
PSD 5	13.09	6		−1		13		0		5		9		−1		−2
PSD 6	11.14	6		4		31		2		11		13		1		2
PSD 7	7.86	15		1		64		−7		14		6		−5		2
PSD 8	3.45	41		5		239		−11		26		41		−6		8

## 4 Discussion

In this study, we demonstrated that both a clinical 2.5D-based motion manager and a 3D-based research motion manager could effectively be used in an MRI-guided MLC tracking research workflow on the 1.5 T MR-linac to mitigate translational prostate motion. For a 2-fraction prostate SBRT delivery, our findings show that both MRI-guided MLC tracking workflows could compensate for linear drift and patient-derived prostate motion, accurately restoring the TPS dose.

The signal latency of the clinical 2.5D-based motion manager was substantially lower (0.6 s) than that of the 3D-based motion manager (6.3 s), as expected. This relatively high latency is however unlikely to be a concern given the low dose rate of the 1.5 T MR-linac (approximately 425 MU/min). Similar imaging frequencies (0.05–1.63 Hz) have been used in the literature for intrafractional prostate motion monitoring [9], [31], [39], [40], [41], [42], [43]. Studies have shown that repeated imaging every 60–180 s can be sufficient to correct for the relatively slow prostate drifts [44].

Although the relatively high latency might suggest the potential use of a prediction filter, such filters are primarily recommended for periodic motion [45]. The irregular motion patterns of prostates [46] make accurate prediction challenging [47]. Given that prostate motion occurs on a much slower timescale than the system response time, prediction filters are generally not applied in this context [20], [21], [48], [49].

Despite the induced latency, our dosimetric analysis demonstrates that MLC tracking significantly improves the dose distribution, effectively restoring the TPS dose as shown by high local 2%/2 mm gamma pass-rates (89%–99%) for both the 2.5D and 3D-based tracking workflows. Additionally, MLC tracking substantially reduced hot and cold spots. The PSDs revealed that in cases of severe drift motion, the dose in the low-dose region could more than double, resulting in additional exposure of the surrounding OARs. This risk would be further exacerbated by the clinical trend of applying high-dose boost volumes to the dominant lesion within the prostate [50], [51]. Both MLC tracking workflows effectively mitigated motion reducing the dose discrepancies to only -11% (2.5D) and 26% (3D). Notably, up to 5% of these differences can be attributed to PSD-TPS coregistration errors rather than motion-induced variations, as confirmed by the TPS comparison with the static case. Furthermore, volume averaging effects may contribute to the dose differences, given that this PSD was positioned within a steep dose gradient (41%/mm in the TPS). Importantly, the benefits of MLC tracking extend beyond extreme drift cases; previous studies report that prostate translations >2 mm occur in 72% of cases after 10 min, with translations >5 mm observed in 17% of cases [8]. These discrepancies are expected to increase further with longer beam-on times during ultra-hypofractionated treatments [8], [9], [10], stressing the need for active motion management techniques such as MLC tracking for ultra-hypofractionated prostate SBRT, especially when considering further reductions of PTV margins below the 2 mm used here.

It should be noted that all dosimetric results were achieved with a relatively simple phantom configurations which served as proxy for a real prostate patient. While clinical prostate SBRT plans might show a larger degree of complexity resulting in less conformal segment shapes, or be influenced by different clinical priorities, e.g. more rigorous urethra-sparing, the tracking results are unlikely to be compounded by plan complexity given how much the MLC leaf speed (10 cm/s in this study) exceeds the speed of prostate motion, which is further evidenced by the excellent dosimetric results shown in a clinical prostate tracking trial [52].

Although we initially anticipated that the 2.5D-based workflow would outperform the 3D-based workflow due to its shorter imaging intervals, both methods exhibited similar performance. Deviations in the 2%/2 mm gamma pass rates relative to the TPS were only 2%–3%. This consistency was also reflected by the PSD readings with average deviations of only 8 ± 15% and 2 ± 6% for respectively linear drift and patient-derived motion. The excellent performance of both workflows offers great potential for future applications. The 2.5D-based workflow demonstrates how MLC tracking could be seamlessly integrated into an existing clinical workflow using many already clinically used components. This demonstrates how close MRI-guided MLC tracking could be to clinical implementation, requiring only minor modifications beyond the current clinical workflow. The 3D-based workflow required relatively more research interfaces, as 3D imaging has not yet been included in the clinical software.

The excellent performance of the 3D-based workflow highlights its potential for future MLC tracking applications. While this study focused on translational motion, the prostate also undergoes substantial rotations and deformation [8], [53]. 3D cine-MRI provides translational, rotational, and deformable information about both the target and the surrounding OARs. This additional information could enable advanced motion management strategies, including differential motion compensation for targets and OARs, deformable motion tracking, multi-target motion compensation, and intrafractional replanning [27]. Beyond online motion adaptation, 3D cine-MRI could also enhance (retrospective) dose accumulation accuracy. While 2.5D-based dose reconstruction workflows have been proposed [20], [43], their reliance on static anatomical assumptions limits their ability to account for continuous 3D changes.

Despite the promising results of both workflows, their current temporal resolution limits their applicability to tumor sites that primarily exhibit non-periodic motion [25]. However, the vendor-provided 2.5D cine-MRI-based motion manager also includes a 6 Hz cine-MRI sequence [15], which we previously utilized for MLC tracking of periodically moving targets [54]. Additionally, advancements in fast 3D MRI acquisition and reconstruction techniques, as well as dynamic patient models, may eventually provide a complete real-time volumetric representation of the patient during treatment [55], [56], [57].

In conclusion, our experiments demonstrated the technical feasibility of 2.5D and 3D cine-MRI-based MLC tracking on an MR-linac for 2-fraction prostate radiotherapy, with both motion management strategies achieving comparable dosimetric improvements despite the difference in latency.

## CRediT authorship contribution statement

**Prescilla Uijtewaal:** Conceptualization, Methodology, Software, Validation, Formal analysis, Investigation, Data curation, Writing – original draft, Visualization, Project administration. **Pim T.S. Borman:** Conceptualization, Methodology, Software, Writing – review & editing. **Peter L. Woodhead:** Software. **Hans C.J. de Boer:** Writing – review & editing. **Bas W. Raaymakers:** Conceptualization, Writing – review & editing, Supervision. **Martin F. Fast:** Conceptualization, Methodology, Writing – review & editing, Supervision, Project administration, Funding acquisition.

## Declaration of competing interest

The authors declare the following financial interests/personal relationships which may be considered as potential competing interests: MLC tracking research at UMC Utrecht is conducted under a research agreement with Elekta AB.
